# Atmospheric Interactions and Cardiac Arrhythmias

**DOI:** 10.1289/ehp.1409636

**Published:** 2015-06-01

**Authors:** Viktor Čulić

**Affiliations:** Department of Cardiology, University Hospital Center Split, Split, Croatia

Although plausible pathophysiological mechanisms link air pollution to arrhythmogenesis, among them altered autonomic tone, repolarization abnormalities, oxidative stress, myocardial ischemia, and increased intracardiac pressure (Link and Dockery 2010), definitive conclusions have not been reached as yet. Langrish et al. (2014) analyzed 13 double-blind randomized crossover studies and found no significant risk of arrhythmia attributable to acute controlled exposure to air pollutants. Three issues related to meteorological factors probably either confound or modify the short-term association between air pollution and cardiac arrhythmia.

First, several meteorological elements, including air temperature, atmospheric pressure, relative air moisture, and wind speed and direction, also are implicated in triggering ventricular (Čulić et al. 2004, 2005) and supraventricular (Čulić et al. 2012, 2013) arrhythmias independent of physical and emotional stress. In the short term, those meteorological factors may facilitate arrhythmias in susceptible patients by increasing circulatory load and thromboinflammatory processes (Čulić 2014).

Second, these same meteorological elements substantially influence concentrations of sulfur dioxide, carbon monoxide, nitrogen dioxide, ozone, and suspended particulate matter (Bertaccini et al. 2012; Ilten and Selici 2008; Ito et al. 2007). In addition, the greatest ozone production and pollution results from stable, dry, hot weather with high atmospheric pressure and low wind (Vanos et al. 2014).

Air pollution may increase human vulnerability to the effects of temperature, and temperature extremes, in turn, influence population vulnerability to air pollution (Burkart et al. 2013; Ren et al. 2006). Vanos et al. (2014) reported that cardiovascular and respiratory mortality due to short-term exposure to gaseous air pollutants was significantly modified by weather types and season. Alberdi et al. (1998) reported that both relative air moisture and air temperature are strongly related to daily mortality even after controlling for air pollution and influenza. Keatinge and Donaldson (2001) suggested that prolonged cold weather with less wind and rain may produce false associations between mortality and certain air pollutants.

Finally, strong mutual interrelations exist among the above-mentioned meteorological elements. Alberdi et al. (1998) pointed out the strong inverse association they observed between relative air moisture and air temperature as an important problem for regression analysis.

Langrish et al. (2014) caution against definitive acceptance of air pollution as an independent trigger of cardiac arrhythmias. However, the studies included in their analysis had no data on meteorological factors. It is likely that interactive effects among air pollutants and meteorological elements bias each other’s association with arrhythmias and other acute cardiac events. Therefore, further research of the health effects of atmospheric factors should continue in order to identify potentially harmful influences for the population as whole as well as for its vulnerable subgroups.

## References

[r1] Alberdi JC Díaz J Montero JC Mirón I 1998 Daily mortality in Madrid community 1986-1992: relationship with meteorological variables. Eur J Epidemiol 14 6 571 578; PMID:9794124 10.1023/a:1007498305075

[r2] Bertaccini P, Dukić V, Ignaccolo R. 2012 Modeling the short-term effect of traffic and meteorology on air pollution in Turin with generalized additive models. Adv Meteorol, article ID 609328; 10.1155/2012/609328

[r3] Burkart K Canário P Breitner S Schneider A Scherber K Andrade H 2013 Interactive short-term effects of equivalent temperature and air pollution on human mortality in Berlin and Lisbon. Environ Pollut 183 54 63; 10.1016/j.envpol.2013.06.002 23941745

[r4] Čulić V Eterović D Mirić D Giunio L Lukin A Fabijanić D 2004 Triggering of ventricular tachycardia by meteorologic and emotional stress: protective effect of ß-blockers and anxiolytics in men and elderly. Am J Epidemiol 160 11 1047 1058; 10.1093/aje/kwh335 15561984

[r5] Čulić V Silić N Mirić D 2005 Triggering of ventricular ectopic beats by emotional, physical and meteorologic stress: role of age, sex, medications, and chronic risk factors. Croat Med J 46 6 894 906; PMID:16342342

[r6] Čulić V Silić N Mirić D 2012 Triggering of supraventricular premature beats. The impact of acute and chronic risk factors. Int J Cardiol 158 1 112 117; 10.1016/j.ijcard.2012.04.059 22592035

[r7] Čulić V Silić N Hodžić M 2013 Triggering of supraventricular tachycardia by physical activity and meteorologic factors. Int J Cardiol 168 4 4295 4300; 10.1016/j.ijcard.2013.04.195 23701934

[r8] Čulić V 2014 Inflammation, coagulation, weather and arrhythmogenesis: is there a linkage? Int J Cardiol 176 1 289 293; 10.1016/j.ijcard.2014.06.078 25037698

[r9] Ilten N Selici AT 2008 Investigating the impacts of some meteorological parameters on air pollution in Balikesir, Turkey. Environ Monit Assess 140 1–3 267 277; 10.1007/s10661-007-9865-1 17955338

[r10] Ito K Thurston GD Silverman RA 2007 Characterization of PM_2.5_, gaseous pollutants, and meteorological interactions in the context of time-series health effects models. J Exp Sci Environ Epidemiol 17 suppl 2 S45 S60; 10.1038/sj.jes.7500627 18079764

[r11] Keatinge W Donaldson G 2001 Mortality related to cold and air pollution in London after allowance for effects of associated weather patterns. Environ Res 86 3 209 216; 10.1006/enrs.2001.4255 11453671

[r12] Langrish JP Watts SJ Hunter AJ Shah ASV Bosson JA Unosson J 2014 Controlled exposures to air pollutants and risk of cardiac arrhythmia. Environ Health Perspect 122 7 747 753; 10.1289/ehp.1307337 24667535 PMC4080532

[r13] Link MS Dockery DW 2010 Air pollution and the triggering of cardiac arrhythmias. Curr Opin Cardiol 25 1 16 22; 10.1097/HCO.0b013e32833358cd 19881339 PMC3750956

[r14] Ren C Williams GM Tong S 2006 Does particulate matter modify the association between temperature and cardiorespiratory diseases? Environ Health Perspect 114 11 1690 1696; 10.1289/ehp.9266 17107854 PMC1665419

[r15] Vanos JK Hebbern C Cakmak S 2014 Risk assessment for cardiovascular and respiratory mortality due to air pollution and synoptic meteorology in 10 Canadian cities. Environ Pollut 185 322 332; 10.1016/j.envpol.2013.11.007 24355413

